# Attribution Analysis of Runoff Change in Min-Tuo River Basin based on SWAT model simulations, China

**DOI:** 10.1038/s41598-020-59659-z

**Published:** 2020-02-19

**Authors:** Jian Hu, Jie Ma, Chao Nie, Lianqing Xue, Yang Zhang, Fuquan Ni, Yu Deng, Jinshan Liu, Dengke Zhou, Linhuan Li, Zhigang Wang

**Affiliations:** 1 College of Water Conservancy and Hydropower Engineering, Sichuan Agricultural University, Ya’an, 625014 P.R. China; 2 Hydrology and Water Resources College, Hohai University, Nanjing, 210098 P.R. China; 3 China Mobile Group Sichuan Company Limited, Chengdu, 610000 P.R. China; 4 Sichuan Shu Tong Geotechnical Engineering Company, Chengdu, 610000 P.R. China

**Keywords:** Hydrology, Environmental impact, Hydrology

## Abstract

To consummate watershed data and better quantify the impact of climate changes and human activities on runoff, we examined the changes and response mechanisms of runoff in the Min–Tuo River Basin, China. In the examination, the Soil and Water Assessment Tool (SWAT) model was used to simulate possible evapotranspiration, actual evapotranspiration, and runoff in 1980, 1990, 1995, 2000, 2005, 2010, and 2015 under different land-use conditions. SWAT weather generator was used to supplement the missing meteorological data. This study presents a quantitative analysis of the climatic and anthropogenic factors contributing to the runoff alteration in the Min–Tuo River Basin using the Budyko methods. The results suggested that the reduced precipitation was the main cause of runoff reduction. The contributions of precipitation, possible evapotranspiration, and underlying surface alterationsof runoff were 56.18%, 37.08%, and 6.74%, respectively. Sensitivity analysis indicated that the runoff alteration was most sensitive to changes of landscape parameters. The aridity index and all the elasticities showed a spatial variations in the Min–Tuo River Basin. The influence of the three factors on runoff reduction varied with seasons. During the high-flow period, changes of the precipitation and possible evapotranspiration and underlying surface had the greatest effect on runoff reduction, while changes of underlying surfaces had the least effect.

## Introduction

There are many physical and biological factors affecting runoff, such as climate change and alterations of the ground surface caused by human activities. The analysis of how these factors influence hydrological processes, particularly runoff, has become a focus of researches on global climate change^1–5^, and an indispensable task for effective management on water resources in River Basins^6^. The studies of the complexity of impacts of climate change and human activities on runoff help for our understanding of runoff changes, improvement on management decisions-makinng in arid areas and finding solution the problem of water shortage^7,8^. While many of the researches lay focus on precipitation, researches on evapotranspiration fail to draw adequate attention, since it has important effect on the hydrologic cycle. Accordingly, the accurate estimation of evapotranspiration in riverine catchments and the sub-basins is an important component of runoff prediction.

Currently, in predicting runoff, and changes in runoff with climatic or landscape alterations, the main methods include statistical approaches, annual water balance methods, and quantitative versus empirical models^9^. These methods have their respective advantages and disadvantages. The statistical (empirical) models are simple, but require long period data collection. Annual water balance methods take into account the overall changes in land use, but not its spatial distribution. They are plagued, then, by the fact that changes in land use, precipitation, and evapotranspiration vary spatially throughout a catchment. With the improvement of human resource management, it is a challenge to quantify the main hydrological changes (e.g. actual evapotranspiration or streamflow)^8,10^. The impacts of human activities and climate change on runoff can be divided into two categories: the first is, on the basis of water balance, the separation of the two elements by determining the actual evapotranspiration; the second is, on the basis of water energy transfer, the summarization of the changes in streamflow^11^. In the application of these two methods, there is the need to estimate the actual evapotranspiration, while the parameters needed often change with climate, soils and vegetation, so it is difficult to quantify them^8,10^. In the calculation of the actual evapotranspiration, SWAT takes these factors into account. The inputs provided by the calibrated SWAT models are more accurate and more reliable. Hydrological models can be used to interpolate missing data, with a relatively high degree of accuracy, and by considering the spatial variations of factors controlling runoff.

Current researches, rely on monitoring data, but there is no recourse for sub-basins with missing data. Usually, the entire basin is used to replace the missing data for a specific sub-basin. It is extremely crucial to achieve a transformation of predicted runoff values from the scale of the entire basin to the sub-basin. The SWAT model is a spatially distributed hydrological model taking into account the integrated effects of these factors. Therefore, SWAT simulations are important in solving the problem of missing data in certain River Basins^12,13^. SWAT can calculate annual precipitation, annual runoff, annual actual evapotranspiration volume and possible evapotranspiration needed in the Budyko’s water-heat coupling equation^14^. These parameters are precisely characterized by the SWAT model. In the analysis of the control parameters in water-heat coupling in a River Basin, traditional methods can only quantitate the influence of climate changes and human activities on runoff at the annual scale. In addition, they overestimate the effects of vegetation changes on runoff, and overlook the intra-annual distribution of climate change and human activities^15^. The seasonal weakening of intra-annual precipitation significantly contributes to changes in runoff, but the effects of such seasonal precipitation changes may differ during the high-flow, normal-flow, and low-flow period^12^. Therefore, runoff changes are also greatly affected by seasonal precipitation changes^16,17^.

The primary objective of this study is to document and simulate the spatiotemporal variations of runoff within the Min-Tuo River Basin using the Budyko water-heat coupling equation^18^ as a framework. At present, the Min-Tuo River Basinis experiencing some problems, such as the unbalanced spatial-temporal distribution of water resources, annual great variation of streamflow, serious water pollution in urban reaches, resulting in degradation of its aquatic ecosystems and destruction to aquatic organisms diversity^9,12^. Though some scholars have made great achievements in this research field, some subbasins in Min-Tuo River Basin still lack hydrological stations or hydrological data. Therefore, the adoption of the SWAT model to generated necessary but missing factors for the Budyko method can facilitate the analysis of runoff changes in these areas and help for water management and control. Inherent in the analysis is an assessment of landscape and seasonal climatic^19,20^ controls on runoff by conducting an attribution analysis of the changes in simulated runoff. To enhance our comprehension of the impacts of climatic and anthropogenic factors on streamflow alteration in the Min-Tuo River Basin, the aims of this study are: (1) to examine the association between these controlling factors and their temporal scale for an attribution analysis of the simulated runoff changes; (2) to make an accurate elucidation of the changes of annual allocation, and conduct a quantitative analysis of the runoff changes in different sub-basins. Eventually, this study will hopefully promote resonable water resource allocation and regulation under changing conditions.

Guo *et al*.^21^ employed Budyko’s assumption for prediction of future runoff changes in the Yangtze River Basin during the 2020s, 2050s, and 2080s. After an attribution analysis of historical runoff changes, they concluded that the relative runoff changes are mainly determined by the changes of precipitation. Du^22^ carried out a correlation analysis of annual runoff and meteorological factors in the Minjiang River Basin during the previous 52 years (1961–2012), finding out that regional precipitation is the main factor affecting runoff reductions in the Minjiang River Basin. Jiang^23^ analyzed runoff changes of Tuojiang River during the second half of the 20th century, finding out that the runoff changes of runoff in the Tuojiang River were greatly affected by synchronous changes of meteorological factors in the river basin. The effects of human activities on runoff in Tuojiang River Basin were minimal. These results are consistent with the results in this paper.

## Results

### Model examination

**Figure 1:**
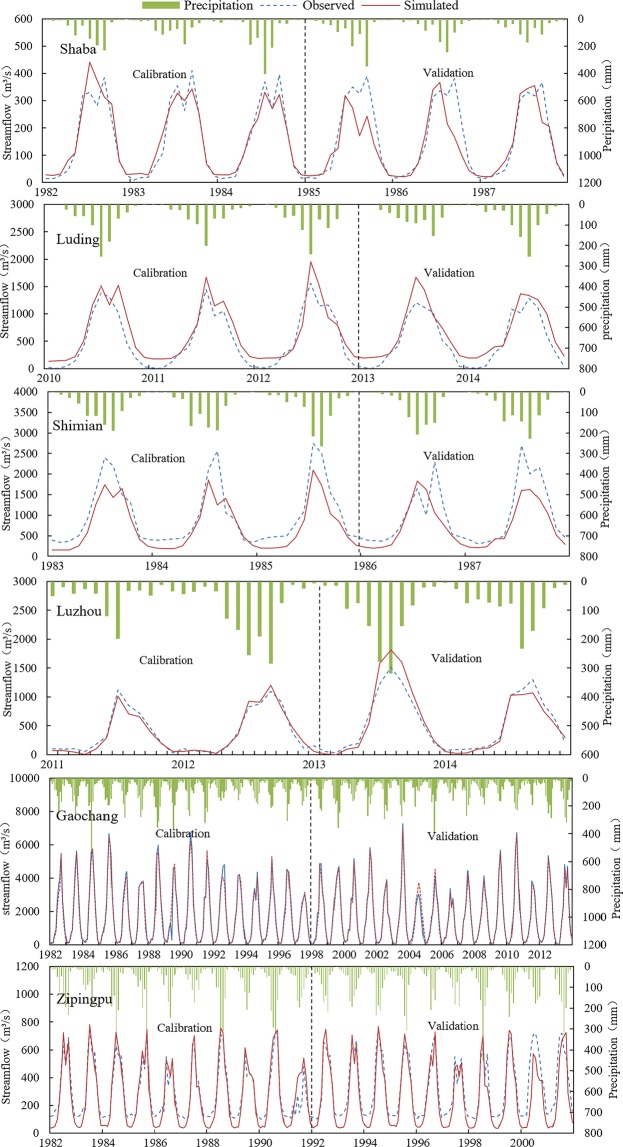
Time series of simulated and observed monthly streamflow with respect to precipitation (P) at Shaba, Luding, Shimian, Luzhou, Gaochang, and Zipingpu gaging stations during the calibration and vadidation periods.

To assess the performance of the model during calibration and validation, simulated and observed monthly flows were used for graphical representation and statistical analysis (Table 1). Figure 1 indicates the comparison between simulated monthly runoff and measurements at six hydrologic stations (Shaba, Luding, Shimian, Luzhou, Gaochang, and Zipingpu). The graphs show that the simulated and observed monthly runoff are in line with most of the reporting periods, and P pattern is used to show the changes.

**Table 1:** The rate determination and verification of the model.

Hydrological station	Phase	Year	R^2^	NSE
Shaba	Calibration	1982~1984	0.92	0.92
	Validation	1985~1987	0.78	0.75
Luding	Calibration	2010~2012	0.90	0.82
	Validation	2013~2014	0.90	0.78
Shimian	Calibration	1983~1985	0.87	0.71
	Validation	1986~1987	0.73	0.61
Luzhou	Calibration	2011~2012	0.76	0.75
	Validation	2013~2014	0.70	0.67
Gaochang	Calibration	1982~1998	0.76	0.76
	Validation	1999~2013	0.90	0.89
Zipingpu	Calibration	1982~1992	0.93	0.87
	Validation	1993~2001	0.88	0.76

SWAT-CUP (SUFI-2) were used for calibration and verification of the SWAT parameters, and after constant adjustment to the parameters, quite good simulation results were obtained (Table 2 is the list of the parameters and their ranges of model calibration). The simulation results showed that the R^2^ and NSE of the Gaochang station at the calibration period were 0.76 and 0.76, respectively, and 0.90 and 0.89, respectively, during the validation period. The values of R^2^ and NSE at Zipingpu gage were both greater than 0.75. Table 1 is a list of the results from the other four hydrological stations. The representative Gaochang and Zipingpu gaging sation are located at the outlet of the Basin and the main stream of the Minjiang River, respectively, according to the standard set out by Moriasi^17^, and the results of the model from all hydrological stations are considered valid during the calibration and validation period.

**Table 2:** The parameters and their ranges for model calibration.

Parameter	Sensitivity ranking	Ranges	Best value
CANMX	1	(−10, 10)	−8.01
SOL_BD	2	(0.08, 0.26)	0.25
SOL_Z	3	(−0.46, −0.12)	−0.14
SOL_K	4	(−0.06, 0.11)	0.05
ESCO	5	(0.76, 1.27)	1.22
SLSUBBSN	6	(0.09, 0.38)	0.21
CH_K2	7	(41.25, 60.51)	44.81
EPCO	8	(−0.42, −0.32)	−0.39
CN2	9	(0.06, 0.17)	0.12
SURLAG	10	(19.55, 29.12)	22.47
CH_N2	11	(0.05, 0.10)	0.08
GW_DELAY	12	(458.76, 548.41)	502.06

### Runoff variation trend

**Figure 2:**
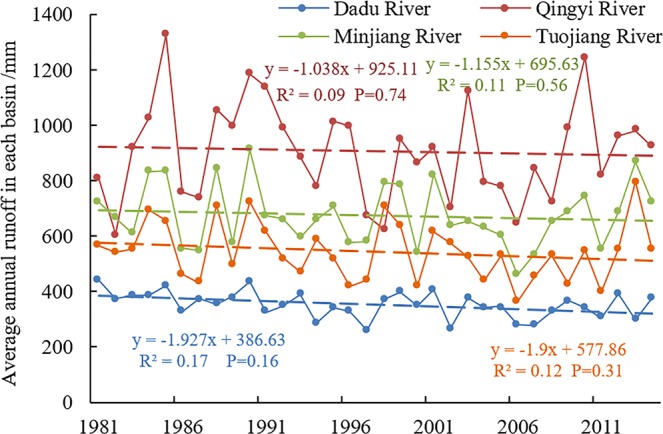
Average annual runoff in each of the major sub-basins between 1981 and 2014.

Figure 2 shows the temporal runoff variations of river basins seleted from 1981 to 2014 based on SWAT simulations The runoff of the four river basins shows a decreasing trend over the 34 year period. the runoff reduction of Dadu River and Tuojiang River basins were relatively substantial, with gradients of −1.93 mm/a (R^2^ = 0.17) and −1.90 mm/a (R^2^ = 0.12), respectively, while the reduction of Qingyi River and Minjiang River basins was relatively less, which gradients −1.04 mm/a (R^2^ = 0.09) and −1.16 mm/a (R^2^ = 0.11), respectively.

**Figure 3:**
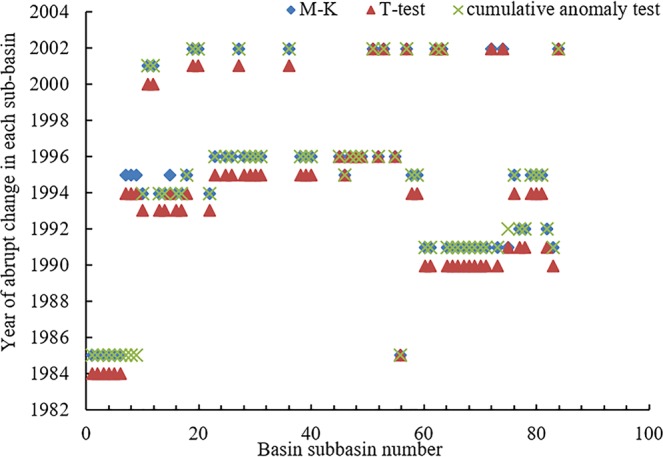
Year of abrupt change in each sub-basin.

M-K method, the sliding T test and the cumulative anomaly test were adopted to study the variations and abrupt changes of SWAT simulated runoff of the Min–Tuo River Basin through time. First, M-K method was used to quantitate runoff of the different sub-basins of Min–Tuo River Basin from 1981 to 2014, and to determine the change points of the time series of runoff of Min–Tuo River Basin. Then, sliding T-test and cumulative anomaly test were employed to determine the time when abrupt changes of runoff occurred within the time series. The results of three methods were consistent when identifying abrupt changes of the sub-basins. Of the 89 sub-basins, 34, 7, 16 and 18 sub-basins underwent significant abrupt-changes in 1993–1996, 1984, 1990–1991 and 2001–2002, respectively (Fig. 3), while the rest 18 sub-basins did not. From the 1990s to 2000s, more than 50 cascade hydropower stations had been constructed on the tributaries of Min-Tuo River Basin, so the reservoirs might have some impact on the runoff. Since these hydropower stations were constructed in different times, the time of abrupt-change occurrence in 89 sub-basins was different^24^. Variability in land use, precipitation, and other meteorological factors might also contribute to the differences, given the complex and diverse terrains (high plateaus and mountains on the west and low basins and hills on the east)^25–28^. In order to facilitate comparison and analysis, simplified and unified time period was set: 1981–1994 was considered the baseline period; 1995 the abrupt-change year, and 1995–2014 the effect period.

### The sensitivity of streamflow alterations to P, ETP, n

**Figure 4:**
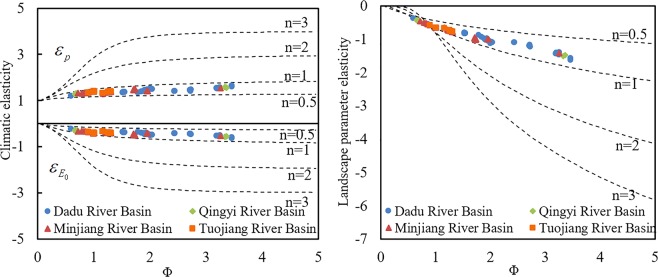
The relationships between elastic coefficient of each factor and aridity index, underlying surface parameters. (**a**,**b**) Represent Climatic elasticity, Landscape parameter elasticity, respectively(Note: blue, green, red and orange represent the elastic coefficients of all factors in the Dadu, Qingyi, Minjiang and Tuojiang River Basin, respectively).

Figure 4 shows the elastic variations of climate and landscape characteristics. The absolute elasticity value for a river basin reflects the sensitivity of runoff in its basin to that factor, including the aridity index (the aridity index was defined as E_0_/P; the arid area is defined when the aridity index is greater than 1^29^) and the landscape parameters. The figures with dotted lines represent the theoretical values of different factors, namely as the aridity index changes. The distribution of precipitation elasticity (ε_p_) of the study site is between 1.27 and 1.64, whereas the distribution of elasticity of possible evapotranspiration (ε_E0_) is between −0.64 and −0.21. The landscape elasticity is between −1.64 and −0.36. The results showed that the absolute elasticity values of precipitation and possible evapotranspiration increase with the increase of the aridity index, but the levels were off when the aridity index increased to a certain value (Fig. 4a). The absolute elasticity value for landscape increased continuously as the aridity index increased (Fig. 4b). This showed that the drier the region, the more sensitive runoff is to changes in the elasticities of precipitation and possible evapotranspiration and landscape. The absolute elasticity values of different factors increase with the increase of landscape parameter, n.

**Figure 5:**
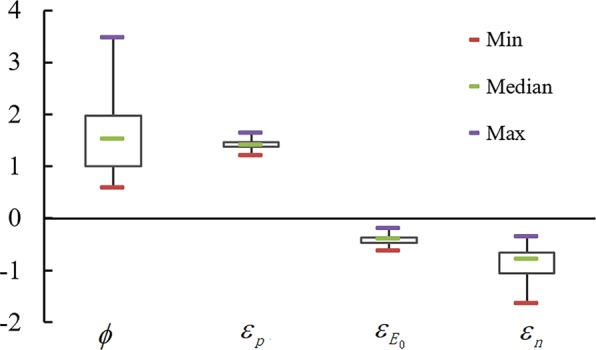
Aridity index and the elasticities of runoff.

Figure 5 showes the distribution of the aridity index and elasticity of different factors in the various sub-basins. Of the 89 sub-basins in the study area, 75% possess an aridity index lower than 2; only 11.2% have an aridity index greater than 3. These data show that most of the study basins are characterized by a humid or semi-humid climatic regime. The precipitation elasticity is concentrated between 1.37 and 1.46, the elasticity of possible evapotranspiration between −0.46 and −0.37, and the elasticity of landscape between −1.05 and −0.65.

**Figure 6:**
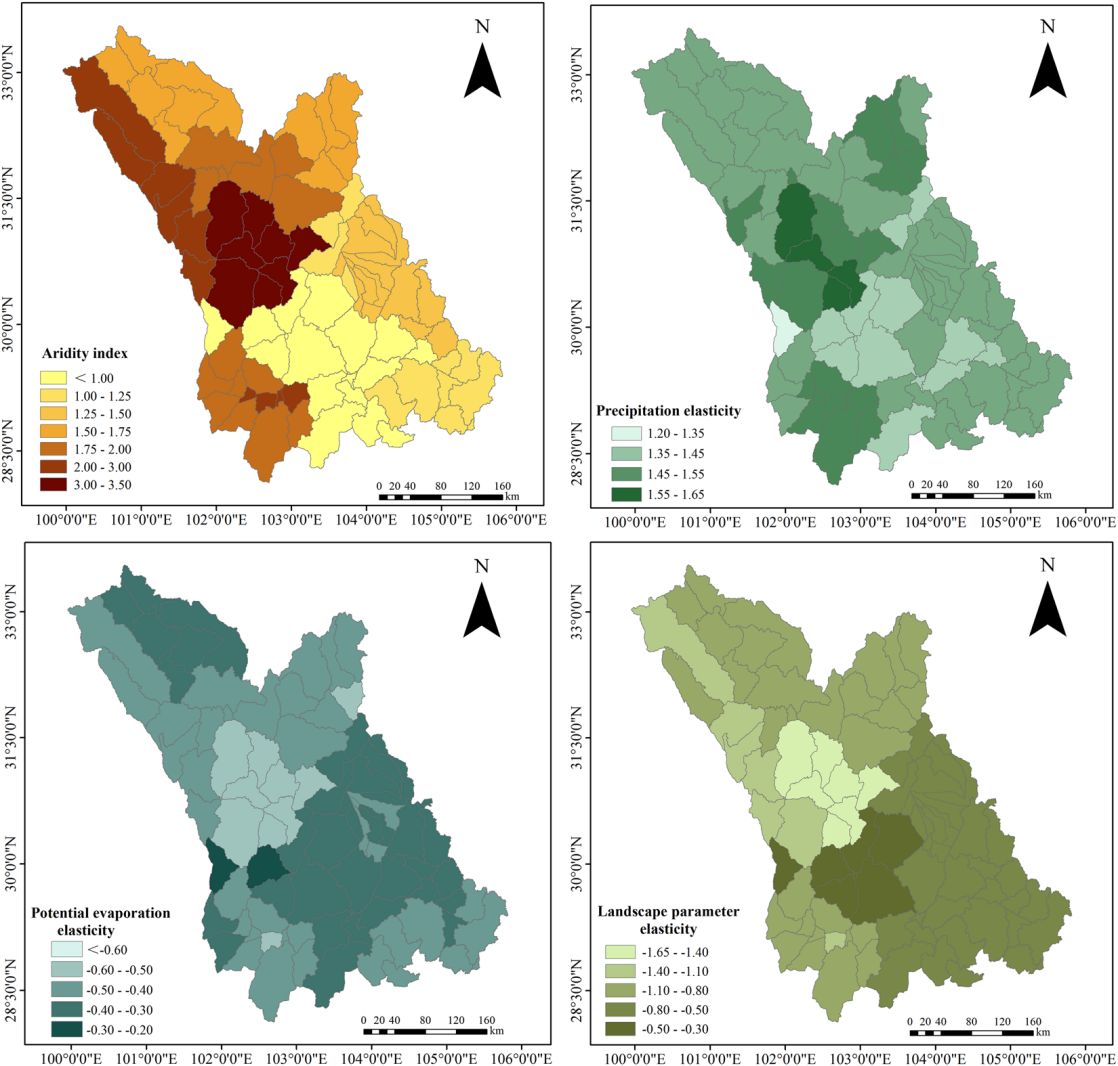
Maps showing the aridity index and elastic coefficient for each factor; (**a**,**b**,**c**,**d**) Reperesent the aridity index, precipitation elasticity, possible evapotranspiration elasticity and landscape parameter elasticity, respectively. (Note: the maps were generated with data available from the Chinese Geospatial Data Cloud using Matlab (version R2016a; https://cn.mathworks.com/)).

The spatial variations in sensitivity of alteration runoff in the different basins is shown in Fig. 6. Qingyi River Basin and the lower reaches of Minjiang River Basin are in humid regions. The sensitivity and absolute values of the elasticity are lower in these basins than in other basins. The aridity indices of some sub-basins in upper reaches of Dadu River and the upper reaches of Minjiang River are greater than 2.5. These basins lie in arid river valleys, so the runoff is more sensitive to changes in the various factors.

### Attribution analysis of runoff interannual variation

**Figure 7:**
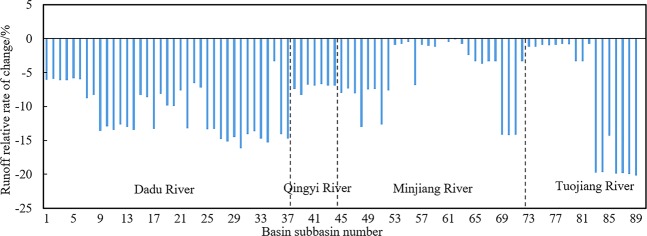
Relative percent change in runoff within each major sub-basin.

Figure 7 shows the relative change when the runoff in the 89 sub-basins between 1995 and 2014 was compared with the runoff between 1981 and 1994. With a few exceptions, the runoff in sub-basins within the entire Min–Tuo River catchment decreased. The runoff reduction was the greatest (>20%) within the lower reaches of Tuojiang River. The overall reduction in Dadu River Basin, Qingyi River Basin, and the upper and middle reaches of Minjiang River were between 5% and 20%. The changes in runoff in the middle reaches of Minjiang River, and the upper and middle reaches of Tuojiang River were minimal, less than 5%.

**Figure 8:**
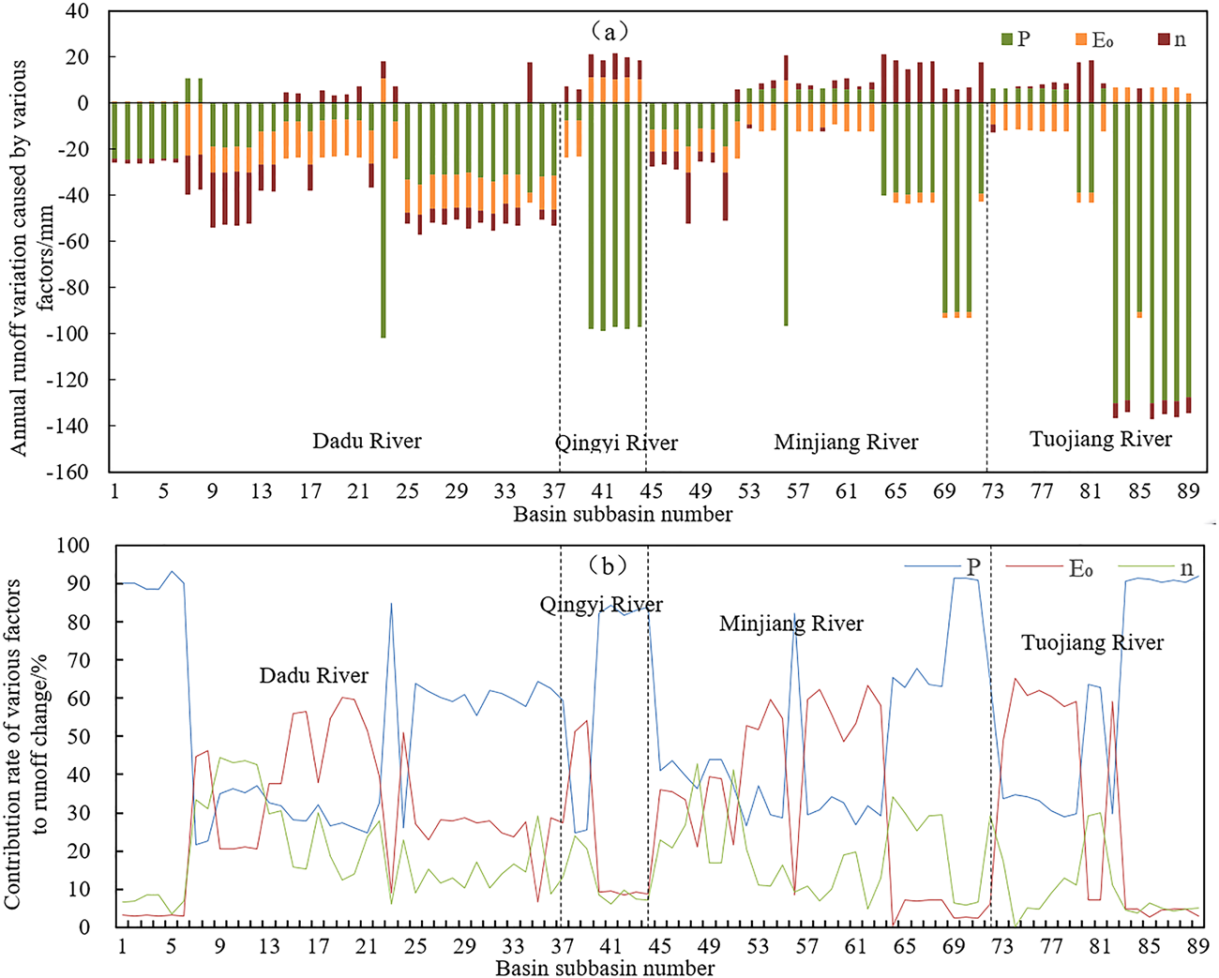
(**a**) Change in annual runoff within the sub-basins; colors refer to contribution of the three factors to the total change); (**b**) Percent contribution to the change in runoff by the three examined factors within each basin (abscissa denotes 89 sub-basins).

Figure 8 shows the results of the attribution analysis. It determines the role of changing precipitation, possible evapotranspiration, and n on the runoff changes in Dadu, Qingyi, Minjiang, and Tuojiang River Basins. Figure 8 shows the runoff changes in the different basins when the effect period and baseline period were compared and an analysis of the causes of runoff changes was made. Precipitation alterations were the main cause of runoff reduction in most sub-basins; possible evapotranspiration changes contributed little to the runoff reduction. In contrast, the changes of the landscape paramater caused runoff increase in some sub-basins, but runoff decrease in other sub-basins (Fig. 8a). In almost all of the sub-basins, runoff decreased (Fig. 8b). The runoff reductions of within 56.18% of these sub-basins were dominated by precipitation changes; precipitation alterations contributed more than 50% of the runoff change in 49.94% of sub-basins. The contribution of possible evapotranspiration dominated 37.08% of sub-basins, while the contribution to runoff changes caused by landscape changes dominated only 6.74% of sub-basins. Overall, the reduced precipitation is the main factor causing runoff reduction, followed by possible evapotranspiration. The effects of changes in the landscape on reduced runoff were relatively small.

### Analysis of seasonal variation of runoff

**Figure 9:**
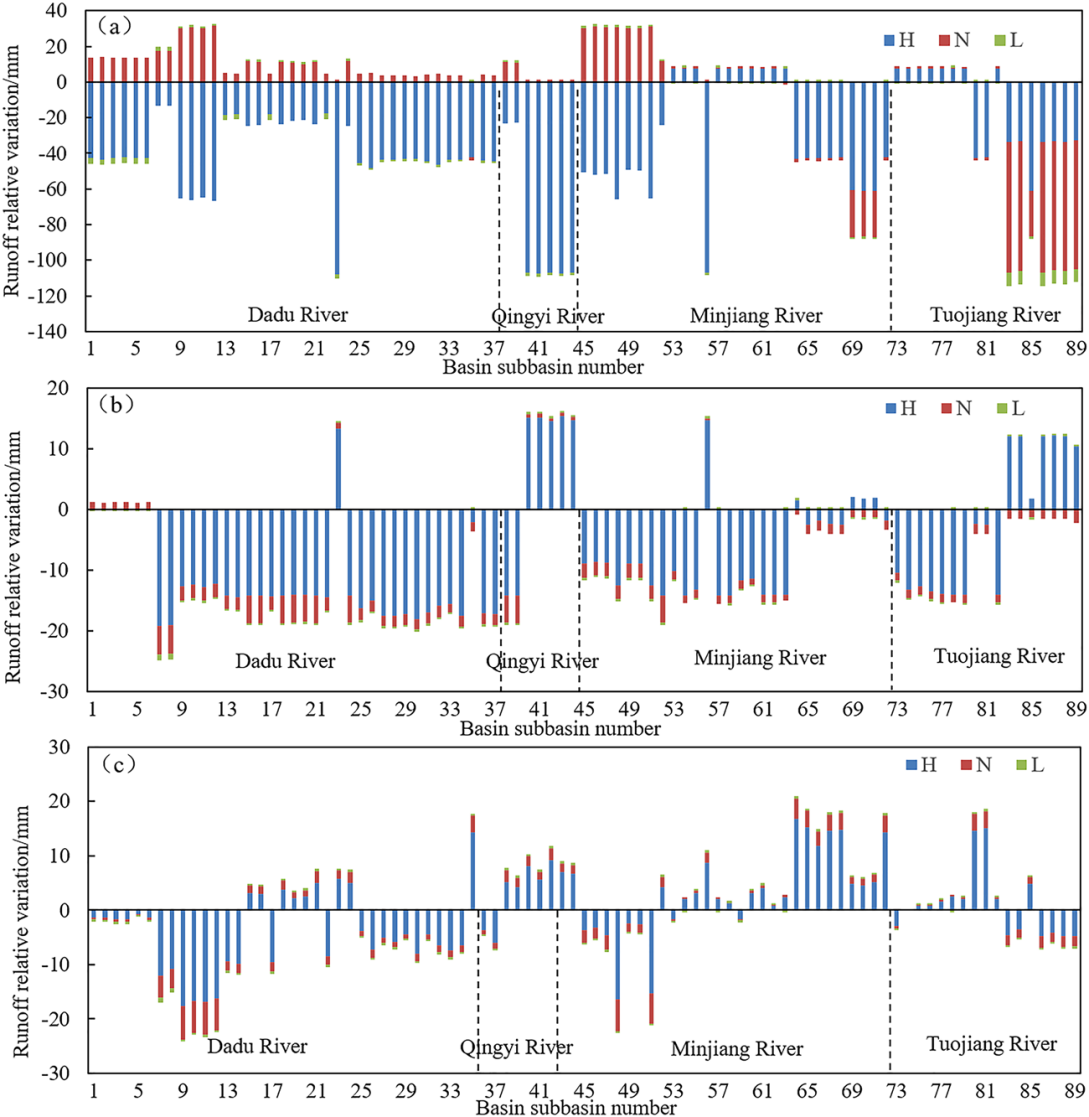
Changes in runoff within the sub-basins to changes in the factors during periods of high flow, normal flow, and low flow. (Note: H, N, L indicate the period of high flow, normal flow and low flow, respectively. (**a**,**b**,**c**) Represent the Changes in runoff within the sub-basins to changes in precipitation, possible evapotranspiration and underlying surfaces, respectively.).

**Figure 10:**
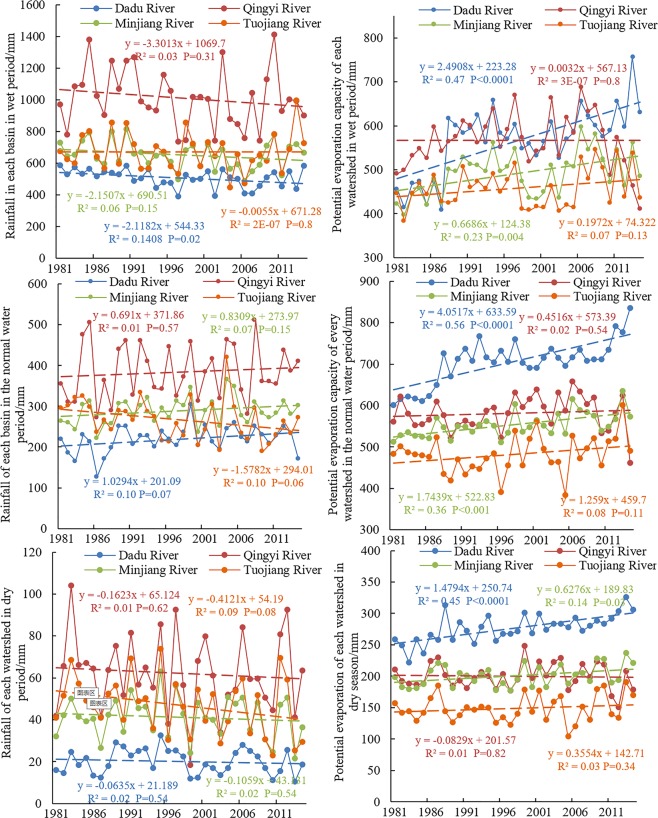
Variation in precipitation (**a**,**b**,**c**) and possible evapotranspiration (**d**,**e**,**f**) within each basin during wet, normal river flow and dry periods.

Figure 9 shows the contributions of the different factors towards runoff changes during the high-flow period, the normal-flow period, and the low-flow period. The control to the different factors on runoff changes varied with seasons. With regards to the entire year, the effects of the various factors on runoff mostly concentrated in the high-flow period. The effects of precipitation on runoff in most sub-basins (79.8%) during the high-flow period resulted in runoff reductions. However, during the normal-flow period, changes in precipitation enhanced runoff in most sub-basins (77.5%). During the low-flow period, the effects of precipitation on runoff were very low. This may be due to significant reductions of precipitation during the high-flow period as a result of climate change within the study area. This particularly applies to Qingyi, Dadu, and Minjiang River basins with precipitation decreases by 3.3 mm/a, 2.12 mm/a, and 2.15 mm/a, respectively. In contrast, reductions within Tuojiang River Basin were not significant. During the normal-flow period, the precipitation in Qingyi, Dadu, and Minjiang river basins showed an increase in precipitation at a rate of 0.69 mm/a, 1.03 mm/a, and 0.83 mm/a, respectively, whereas within Tuojiang River Basin, precipitation decreased at a rate of −1.58 mm/a. This directly causes greater changes of runoff at the lower reaches of Tuojiang River Basin during the normal-flow period than the high-flow period. Although precipitation during the low-flow period also declined, precipitation during the low-flow period only accounted for a small proportion of annual precipitation. Therefore, the effects of precipitation during the low-flow period on runoff was minimal (Fig. 10a). The effects of possible evapotranspiration on runoff in 93.2% of the sub-basins during the high-flow period were more significant than during the normal-flow period and low-flow period. During the high-flow period, the possible evapotranspiration within Dadu, Minjiang, and Tuojiang River basins increased at a rate of 2.49 mm/a, 0.67 mm/a, and 0.20 mm/a, respectively, while evapotranspiration in Qingyi River Basin showed a slight increase (0.003 mm/a). During the normal-flow period, the possible evapotranspiration in Dadu, Qingyi, Minjiang, and Tuojiang River basins increased at a rate of 4.05 mm/a, 0.45 mm/a, 1.74 mm/a, and 1.26 mm/a, respectively. During the low-flow period, the possible evapotranspiration of Dadu, Minjiang, and Tuojiang River basins increased at a rate of 1.48 mm/a, 0.63 mm/a, 0.36 mm/a, while in Qingyi River Basin it decreased at a rate of −0.08 mm/a (Fig. 10b). The possible evapotranspiration in most basins increased during the high-, normal-, and low-flow periods, while runoff changes caused by alterations in possible evapotranspiration varied with seasons. This shows that seasonal variations in precipitation in the study area greatly affected the runoff changes. The effects of landscape changes on runoff during the high-flow period were more significant in 98.9% of sub-basins than during the normal-flow and low-flow periods. Changes in the landscape along with seasonal changes were low. The reason for these differences may be the abundant precipitation during the high-flow period, causing the soil in the landscape to be saturated with water and produce more runoff. During the low-flow and normal-flow periods, the precipitation was lower with higher evapotranspiration and infiltration and the lower runoff.

## Discussion and Conclusions

In this paper, the SWAT model and the Budyko model were used for systematic evaluation of the effects of seasonal climate changes and landscape changes on runoff in 89 sub-basins in the Min–Tuo River Basin. Budyko water–heat coupling equation and the river basin water balance equation were used to calculate the elasticities of precipitation, possible evapotranspiration, and landscape. Quantitation was made concerning the contributions of these three factors to the runoff changes and the following conclusions were drawn: (1) within the 89 sub-basins selected in this paper, the mean annual runoff during the effect period (1996–2014) significantly decreased in most sub-basins compared with the baseline period. The contributions of three examined factors towards runoff changes exhibited seasonal differences. During the high-flow period, the contributions of the three different factors to runoff changes are clearly dominant; (2) Most of the selected river basins in this paper have a humid or semi-humid climate with a minority in arid river valleys. The contributions of the different factors towards runoff changes were spatially variable. The runoff in dry river valleys was more sensitive to changes in the different factors compared with that in humid regions; (3) Attribution analysis of the changes of runoff during the baseline period (1981–1994) and the effect period showed that climate change (reduced precipitation and increased evaporation) are the main factors causing reduced runoff. Runoff changes due to changes in underlying surfaces show varied differences in different regions.

**Figure 11:**
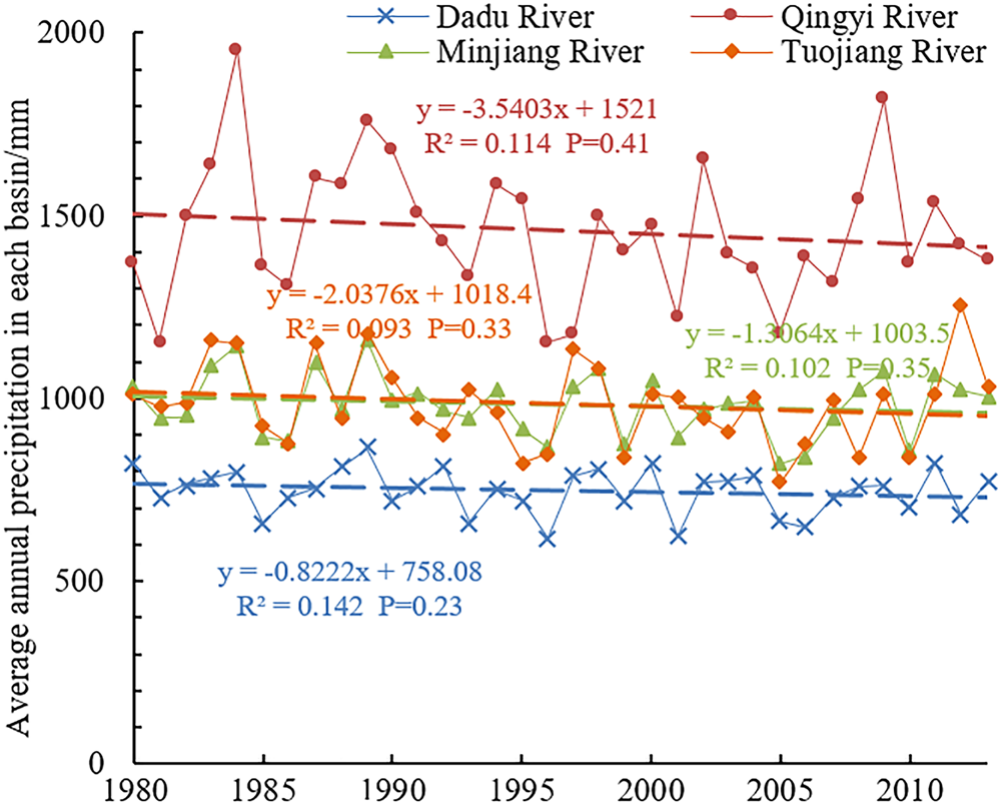
Average annual rainfall in each basin between 1981 and 2014.

From 1981 to 2014, the mean annual precipitation of the Dadu River Basin was 746.44 mm at a decrease at a rate of −0.82 mm/a. The mean annual precipitation within Qingyi River Basin was 1458.15 mm at a decrease rate of −3.54 mm/a. The mean annual precipitation of the Minjiang River Basin was 982.66 mm at a decrease rate of −1.31 mm/a; and the mean annual precipitation of the Tuojiang River Basin was 984.55 mm at a decrease rate of −2.04 mm/a (Fig. 11). They are consistent with the runoff variations shown in Fig. 2. Similarly, seasonal changes of precipitation within the study area were great, but variable. Precipitation significantly decreased during the high-flow period, and increased during the normal-flow period, but did not change during the low-flow period. The causes of reduced precipitation are complex, but related to natural climate fluctuations and the effects of human activities^25^. The study site is located in the mid-latitudes of Eurasia. Climate changes in this region are simultaneously affected by atmospheric circulation (such as Siberian warm and humid air current) and oceanic circulation (such as the Pacific Ocean warm and humid air current)^26^. In addition, the weakening of the East Asia summer monsoon is the main reason for reduced precipitation in the study area^23^. Table 3 shows the minor changes in the percent of cultivated land and forests between 1980 and 2015, with decreased of 3.79% and 0.28%, respectively. However, grassland area increased by 1.31% and urban residential land by 111.99% as a result of rapid economic development in southwest China, particularly after 2000. The resultant emission of greenhouse gases and atmospheric aerosols has led to global climate change, changes in land use, and urbanization. This resulted in the heat island effect affecting precipitation changes to varied degrees, thereby indirectly reducing runoff^28^. In addition, the construction of cascade reservoirs, water diversion and irrigation works, and soil and water conservation measures have changed the landscape, in turn causing runoff reduction^30^.

**Table 3:** Land use and vegetation cover changes in the MinTuo river basin in 1980, 1990, 1995, 2000, 2005, 2010 and 2015.

year	Farmland		Forestland		Grassland		Water		Snow		Residential land		Unulitized land	
	area (km^2^)	rat (%)	area (km^2^)	rat (%)	area (km^2^)	rat (%)	area (km^2^)	rat (%)	area (km^2^)	rat (%)	area (km^2^)	rat (%)	area (km^2^)	rat (%)
1980	43161		55875		58327		1028		347		1209		3013	
1990	43215	0.13	56268	0.70	57612	−1.23	977	−4.96	329	−5.19	1610	33.17	2949	−2.12
1995	42850	−0.72	57378	2.69	56844	−2.54	970	−5.64	333	−4.03	1776	46.90	2806	−6.87
2000	42710	−1.04	55695	−0.32	58493	0.28	1047	1.85	330	−4.90	1959	62.03	2715	−9.89
2005	42139	−2.37	55546	−0.59	58808	0.82	1038	0.97	329	−5.19	2357	94.95	2714	−9.92
2010	41993	−2.71	55595	−0.50	58730	0.69	1076	4.67	329	−5.19	2459	103.39	2778	−7.80
2015	41525	−3.79	55716	−0.28	59091	1.31	1172	14.01	333	−4.03	2563	111.99	2560	−15.03

Climate change due to natural causes is an uncontrollable factor. Zhao *et al*. documented that climate change and water-carbon coupling cycles were closely related^31^. However, saving energy and reducing emissions are effective measures to alleviate global warming. These measures include effective control on overall carbon emissions and further strengthening of the controls on non-carbon dioxide greenhouse gas emissions, accelerating the development of non-fossil fuel energy sources, stable development of wind power, accelerating the development of solar power, promoting low-carbon urbanization and advocating low-carbon lifestyle. It should be noted that changes in landscape management measures and land use had resulted in increased water consumption^32^. Therefore, in the future development, importance should be attached to the human-nature harmony.

Quantifying the impacts of climate and human activities on streamflow has become a central theme in climate and hydrological research. However, the factors contributing to climate change and human activities in different regions differ because of the adoption of different methods and baseline periods. Whereas, in different areas, the impact factors were distinctive due to the application of distinct methods and baseline periods. And this paper provides a more detailed analysis of the spatial variations in runoff and the factors affecting runoff. The SWAT modeling results were combined with Budyko’s assumption for the attribution analysis of runoff reductions in the Min–Tuo River Basin. Our results showed that reduced precipitation was the main cause of runoff reduction. While previous researched centered on the climate changes affecting the runoff only in southwest China^33^, for instance, Yangtze River^34^, Yinjiang River Watershed^35^, Jinsha River Basin^36^, and Karst catchments^37^. Their results are consistent with ours in this paper. Thus, the analysis provides scientific and technical support for development, utilization, assessment, and optimization of water resource.

Without real evaluation of the uncertainty of the model itself with the defects of the input data and model parameters, the credibility of the model task will be difficult to achieve. Many researches have worked around the uncertainty of parameters in the hydrological modeling. Among these three sources, the most general is the parameter uncertainty, but it can be controlled easily with proper calibrations. The most representative is work done by Zhao *et al*.^38^. Their recent research discussed three uncertainty methods (ParaSol, GLUE, SUFI-2) using distributed hydrological model (SWAT), and SUFI-2 process indicated superiority over the other two processes in the uncertainty analysis. The SWAT Calibration and Uncertainty Programs (SWAT-CUP) (SUFI-2) had been used in calibration and verification of hydrological cycle simulation by more and more Scholars^39–41^. However, the input data of the model only included meteorological data, soil data and land use data, but the reservoir data also have a certain impact on the simulation results. Owing to the limitation of data, reservoir information had not been gathered. In this study, SWAT-CUP (SUFI-2) is used for the calibration and verification of SWAT parameters using recorded data to improve the accuracy of the model.

Wu *et al*. pointed out that quantitative estimations are often based on the hypothesis that climate change and human activities are mutually independent^42,43^. Whereas, in natural areas, the two factors are interrelated and continue to interact at each location^44^. According to Budyko’s hypothesis, the estimation of climate elasticity showed that the change of water content in soil can be ignored over a long period of time. When the elasticity was evaluated as a first-order Taylor expansion approximation, a potential error was revealed by Yang *et al*.^45^. In addition, the climatic factors and human activities had not been completely saparated. Consequently, in this study, we could not quantify the contributions of the specific human activities and every climatic factor. And n reflected the feature of a particular catchment, including the terrain, soil, land use and so on^29^. All these are regarded as the impacts of human activities on runoff. But how these factors affect runoff needs further study.

Certainly all these factors could result in some deviations in the calculation results. The above analysis proved that there are still great difficulties and uncertainties in quantifying the separate impacts of climate change and human activities on streamflow alteration due to complex interactions and limited data (e.g., reservoir information), climate change (such as precipitation, actual evapotranspiration) and increased human activities (such as land use change, reservoir construction). Then, future researches should clearly differentiate the effects of climate change and human activities on streamflow. And the SWAT simulation also has many uncertainties. The accuracy of model simulation should be improved in the future research.

## Materials and Methods

### Study area

At the famous place of the world-Dujiangan Irrigation Area, the part of Minjiang River joins Tuojiang River, so Minjiang River and Tuojiang River are generally referred to as Min-Tuo River for short. Minjiang River is the largest tributary of the Yangtze River and originates in the southern foot hills of the Min Mountains. The river flows from northwest to southeast through the western part of Sichuan Basin. The entire length of the river is 1,279 km; the basin encompasses an area of 135,200 km^2^. The river possesses a large volume of water and merges with important tributaries such as Heishui River, Zagunao River, Dadu River, and Mabian River before joining the Yangtze River at Yibin. Most of Minjiang River Basin is characterized by a subtropical climate. The atmospheric temperature of the main stream gradually increases from the upper reaches to the lower reaches of the river: the mean annual temperature of the river north of the Zhenjiang pass varies between 5 °C and 9 °C; it increases to around 15 °C at the edge of the basin in Dujiangyan City. The precipitation in the region varies significantly seasonally. During the rainy season, particularly between June and September, there are frequent torrential rains. The rainfall during summer (from June to August) and autumn (from September to November) accounts for more than 80% of the annual rainfall. Precipitation also varies spatially within the basin, and is particularly affected by terrain (topography). Dadu River originates along the southern foot of Mount Guoluo in Amne Machin Mountains in Yushu Tibetan Autonomous Prefecture, Qinghai Province. Qingyi River originates in the Western Sichuan Camp in Balang Mountain and Jiajin Mountain within Qionglai Mountains. Both rivers are tributaries of Minjiang River, and they join Minjiang at Leshan.

Tuojiang River is a primary tributary of Yangtze River, and is an economically and ecologically important river passing through the hinterland of the Sichuan Basin. The river originates at Jiuding Shan in Mianzhu City flowing all the ways to the town of Hanwang, passing through the mountains and finally entering the Chengdu Plain. In Chengdu Plain, it converges with the Yangtze River at the city of Luzhou. The length of the river is 627.4 km encompassing a basin area of 27,800 km^2^.

In Min-Tuo River Basin, there are seven main soil types-haplic luvisols, gelic leptosols, cumulic anthrosols, mollic leptosols, dystric cambisols, calcaric regosols and eutric leptosols, accounting for 21.36%, 16.20%, 11.29%, 10.35%, 9.84%, 6.25%, and 4.51% of the basin. The haplic luvisols, gelic leptosols and mollic leptosols mainly cover Dadu River Basin and the upstream of Minjiang River Basin (the central north area and the south-west area), the cumulic anthrosols, dystric cambisols, and calcaric regosols distributes in Qingyi River Basin, Tuojiang River Basin and the downstream area of Minjiang River Basin, and the eutric leptosols distributes along the river channels. There are three major land use types-farmland, forestland, and grassland (Table 3). The farmland is mainly located in low altitude area, the grassland characterized by middle and low coverage with relatively even distribution, and the forestland is mainly located in high altitude area.

### Data

**Figure 12:**
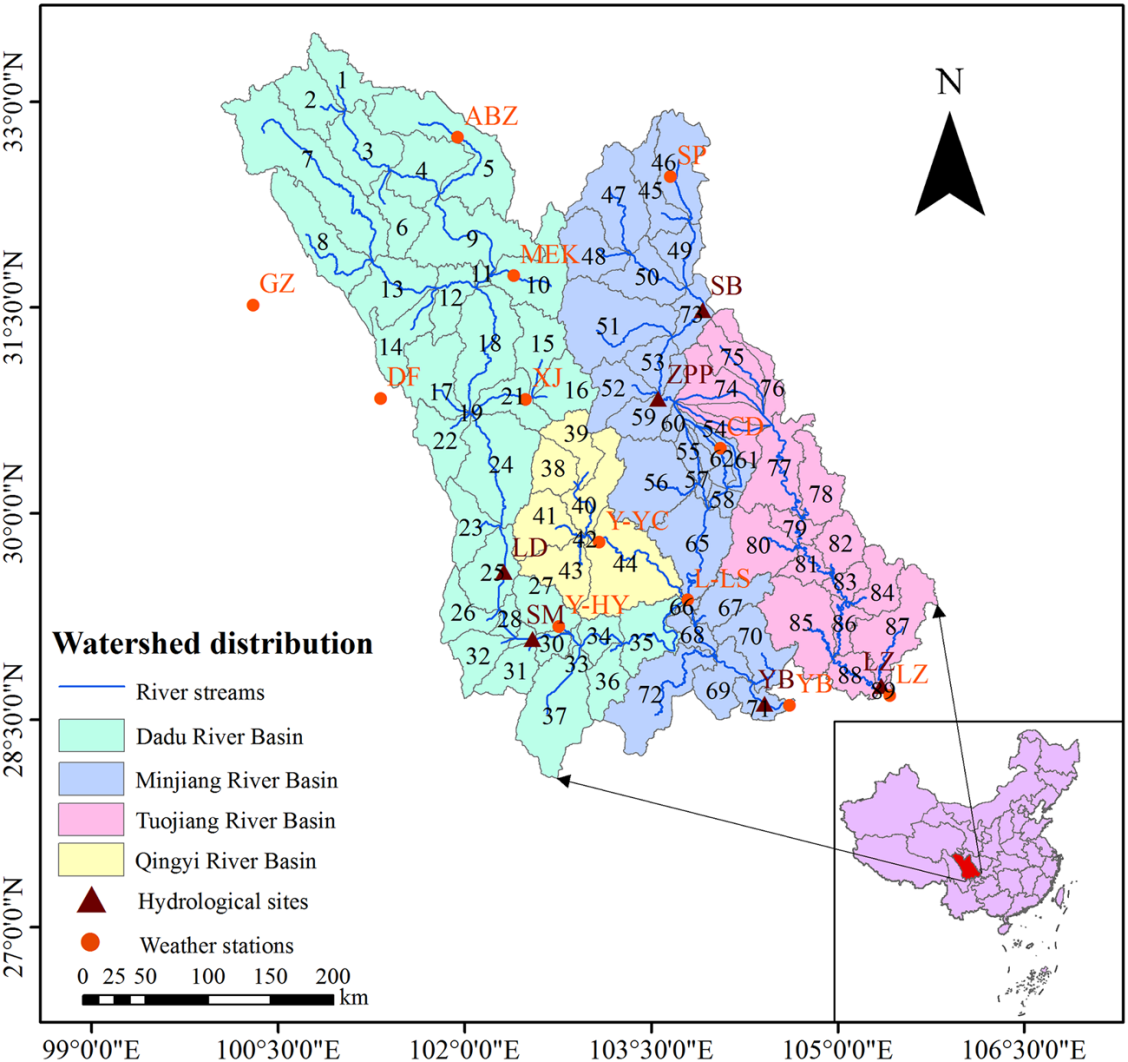
Basin and sub-basin distribution within the study area. (Note: the maps were generated with data available from the Chinese Geospatial Data Cloud using ESRI’s ArcGIS (version 10.1; http://www.gscloud.cn/). And the numbers represent the sub-basin numbers).

For this study, 12 state meteorological stations were selected within Min–Tuo River Basin and its surrounding areas (Fig. 12). Daily meteorological data were collected at the stations between 1981 and 2014, and compiled by the China Meteorological Administration. The collected data include daily precipitation, daily mean temperature, daily maximum and minimum temperature, daily number of sunshine hours, daily mean wind speed, and relative humidity. Measured runoff from 6 hydrological stations (Fig. 12) were also used in this study. Land use data and soil data were obtained from the Heihe data center (Table 4). The CGIAR-CSI SRTM 30 m resolution, digital elevation model database (DEM) was used to extract the boundaries of the sub-basins. The data were selected for model calibration and verification shown in Table 1. The SWAT model dividing the study site into 89 sub-basins (Fig. 12), was constructed^46^. There were 37, 7, 28, and 17 sub-basins in the Dadu, Qingyi, Minjiang, and Tuojiang River Basins, respectively. The precipitation, possible evapotranspiration, and actual evapotranspiration of the different sub-basins obtained from the study site were used for the analysis.

**Table 4:** Data sources and resolution.

Name	Source	Format	Year	Resolution
DEM data	CGIAR-CSISRTM elevation database //srtm.csi.cgiar.org/	GRID	2010	30 m
Land use data	Heihe data center //westdc.westgis.ac.cn/	GRID	1980, 1990, 1995, 2000, 2005, 2010, 2015	1 km
Soil data	Heihe data center //westdc.westgis.ac.cn/	GRID	2010	1 km
Meteorological data	Meteorological stations in the study site	/	1979–2014	Daily
Hydrological data	Hydrological stations in the study site	/	1982–3013	Monthly

## Methods

### SWAT simulation analysis

SUFI-2 algorithm was used for parameter calibration, validation and evaluation of the SWAT model. The measured monthly runoff data were selected for model calibration from 6 major hydrological stations (Shaba, Luding, Shimian, Luzhou, Gaochang, and Zipingpu, Fig. 12). Luding and Gaochang stations are typical outlet stations, located at the mouth of Tuojiang and Minjiang Rivers, respectively.

SWAT model constructed for Min–Tuo River Basin was evaluated by comparing simulated versus observed runoff data using two methods: (1) correlation analyses (coefficient of determination, R^2^), and (2) the Nash-Sutcliffe Efficiency Coefficient (NSE). The NSE measures the goodness-of-fit between the model simulation values and the observed values. Generally, a model is believed satisfactory when R^2^ > 0.7 and NSE > 0.6^47^. Therefore, SWAT model is suitable for application in Min–Tuo River Basin. Besides, from Table 1, the values of R^2^ at six gages are seen higher than 0.7, and NSE higher than 0.6 for both calibration and validation periods. Overall, the simulation results were satisfactory for the subsequent analysis.

### Abrupt change point detection

Abrupt-change analysis methods can be applied to time-series runoff data^48^ to determine the timing of significant, rapid changes of runoff. In this paper, Mann-Kendall (M-K), sliding T, and cumulative anomaly tests were used for obtaining the runoff time series data from the study area to identify abrupt runoff changes. M-K test is a non-parametric statistical test, indepedent of outliers and human factors, so it facilitates calculation. This test finds wide uses in trends analysis for climate change and is recommended by the World Meteorological Organization for trend analysis of environmental data^49–52^. The cumulative anomaly method has a good level of quantitation and certainty, while, the sliding T-test is considered intuitive and simple^53,54^. Given the strengths and weaknesses of each of the methods, all three of them were used for the abrupt-change analysis of the 1981–2014 time series runoff data in the Min–Tuo River Basin to improve the reliability and accuracy of the study results.

### Estimation of the climatic elasticity of runoff and the elasticity coefficient of the underlying surfaces

The water-heat coupled balance equation at the mean annual scale proposed by Budyko^55^ is widely used in water and energy balance studies of large river basins^56,57^. Budyko assumes that as the climate becomes drier, the mean annual actual evapotranspiration in the river basin gradually approaches the mean annual precipitation^58^. Yang *et al*.^29^ derived the following water-heat coupled balance equation for river basins based on Budyko’s assumption:

1 $${E}{=}\frac{{P}{{E}}_{{0}}}{{({{P}}^{{n}}+{{E}}_{{0}}^{{n}})}^{\frac{{1}}{{n}}}}$$

where E is the mean annual actual evapotranspiration, P the mean annual precipitation, E_0_ the mean annual possible evapotranspiration, and n a parameter reflecting the characteristics of the landscape in the River Basin (known as landscape parameter). The descriptions of the landscape in the River Basin include terrain, soil, and vegetation^59^. From the mean annual water-energy balance equation, where R = P − E, we obtain R = *f* (P, E_0_, n). The mean annual evapotranspiration E, possible evapotranspiration (E_0_), and precipitation (P) were substituted into Eq. (1). Calculations of n were carried out using a step length of 0.001. The n value with the lowest error in the equation was used as the n value of the corresponding landscape parameter in the River Basin.

The runoff elasticity^60,61^ is the ratio of the ratio of runoff change to the ratio of change of the different factors.

2 $${\varepsilon }=\frac{\partial {R}}{\partial {X}}\frac{{R}}{{X}}{.}$$

At the national scale (i.e., all of China), changes in the elasticity of changes in runoff to the different factors have been ranked in the following order: precipitation > land use and land cover change (LUCC) > relative humidity > solar radiation > maximum air temperature > wind speed > lowest air temperature^62^. In addition, precipitation and LUCC were determined to be the main factors affecting runoff. In light of these national results, precipitation, possible evapotranspiration, and changes in landscape were considered as the main factors herein controlling changes of runoff. Runoff changes can be expressed as:

3 $$\Delta {R}=({{\varepsilon }}_{{P}}\frac{\Delta {P}}{{P}}+{{\varepsilon }}_{{E}{{T}}_{{0}}}\frac{\Delta {E}{{T}}_{{0}}}{{E}{{T}}_{{0}}}+{{\varepsilon }}_{{n}}\frac{\Delta {n}}{{n}}){R}$$

where, ${{\varepsilon }}_{{p}},{{\varepsilon }}_{{{E}}_{{0}}},{{\varepsilon }}_{{n}}$ represent the elasticities of precipitation, possible evapotranspiration, and landscape, respectively. The elasticities of various factors were derived by combining the Budyko water-heat coupled balance equation and water balance equation.

### Relative ratio of change of runoff and relative contributions of different factors to runoff changes

The relative ratio of runoff change is calculated using the follow equation:

4 $${\eta }=\frac{{{R}}_{{2}}{-}{{R}}_{{1}}}{{{R}}_{{1}}}\times 100 \% .$$

where, R_1_ and R_2_ represent the mean annual runoff during the baseline period and the effect period, respectively.

According to Eq. (3), the effects of different factors on the change of runoff can be obtained this way:

$$\Delta {R}_{x}={\varepsilon }_{{\rm{x}}}\frac{R}{{\rm{x}}}\Delta {\rm{x}}$$

where, R is the mean annual runoff; x one of the factors affecting the change of runoff, including precipitation, possible evapotranspiration, and landscape; εx the elasticities of the different factors affecting the change of runoff; Δx the change of an influencing factor when the effect period is compared with the baseline period; and ΔRx the degree of influence of the corresponding factor on the change of runoff.

### Seasonal effects of different factors on the change of runoff

The Min–Tuo River Basin is characterized by seasonal climate variations. Precipitation during the rainy season (from June to September) accounts for two-thirds of the total annual precipitation. Thus, rainfall is not uniformly distributed throughout the year, but relatively concentrates within a few months. The runoff of the river also concentrates within months from June to September, causing great differences to the maximum and minimum values of monthly runoff. The high-flow period is defined as a period depending on rainfall or snowmelting to recharge the streamflow and the low-flow period as a period mainly based on groundwater to recharge the water source. Due to the differences in river basin morphometry and position, this paper adopted unified time periods: the time from June to September was classified as the high-flow period, the time from December to February as the low-flow period, and the remaining months were classified as the normal-flow period. Budyko’s assumption was then used for attribution analysis of runoff changes during these three time periods with different flows.

## References

[1] Khetrapal N (2018). Human activities and climate change. Encyclopedia of the Anthropocene.

[2] Ayele WT, Ahmed EN, Lars R (2018). & Heinrich Jürgen. Catchment response to climate and land use changes in the upper blue nile sub-basins, ethiopia. Science of The Total Environment.

[3] Mwangi HM, Julich S, Patil SD, Mcdonald MA, Feger KH (2016). Relative contribution of land use change and climate variability on discharge of upper mara river, kenya. Journal of Hydrology: Regional Studies..

[4] Napoli M, Massetti L, Orlandini S (2017). Hydrological response to land use and climate changes in a rural hilly basin in italy. Catena..

[5] Huo Z, Feng S, Kang S, Li W, Chen S (2010). Effect of climate changes and water-related human activities on annual stream flows of the shiyang river basin in arid north-west china. Hydrological Processes..

[6] Wang FY (2019). Partitioning climate and human contributions to changes in mean annual streamflow based on the budyko complementary relationship in the loess plateau, china. Science of the Total Environment..

[7] Khalil AF, McKee M, Kemblowski M, Asefa T (2005). Basin scale water management and forecasting using artificial neural networks. Journal of the American water resources association..

[8] Li X (2018). Hydrological cycle in the heihe river basin and its implication for water resource management in endorheic basins. Journal of Geophysical Research Atmospheres..

[9] Wang SJ, Yan M, Yan YX, Shi CX, He L (2012). Contributions of climate change and human activities to the changes in runoff increment in different sections of the yellow river. Quaternary International..

[10] Li CB (2018). An analytical approach to separate climate and human contributions to basin streamflow variability. Journal of Hydrology..

[11] Bosshard T (2013). Quantifying uncertainty sources in an ensemble of hydrological climate impact projections. Water Resources Research..

[12] Zhou F (2013). Hydrological response to urbanization at different spatio-temporal scales simulated by coupling of clue-s and the swat model in the yangtze river delta region. Journal of Hydrology..

[13] Lin BQ (2015). Analyses of landuse change impacts on catchment runoff using different time indicators based on swat model. Ecological Indicators..

[14] Xue LQ (2017). Identification of potential impacts of climate change and anthropogenic activities on streamflow alterations in the tarim river basin, china. Scientific Reports..

[15] Rumbaur C (2014). Sustainable management of river oases along the tarim river (sumario) in northwest china under conditions of climate change. Earth System Dynamics Discussions..

[16] Meixner T (2016). Implications of projected climate change for groundwater recharge in the western united states. Journal of Hydrology..

[17] Kim U, Kaluarachchi JJ (2009). Climate change impacts on water resources in the upper blue nile river basin, ethiopia. JAWRA Journal of the American Water Resources Association..

[18] Gao M, Chen X, Liu J, Zhang Z (2018). Regionalization of annual runoff characteristics and its indication of co-dependence among hydro-climate–landscape factors in jinghe river basin, china. Stochastic Environmental Research and Risk Assessment..

[19] Wu XL, Xiang XH, Chen X, Zhang X, Hua WJ (2018). Effects of cascade reservoir dams on the streamflow and sediment transport in the wujiang river basin of the yangtze river, china. Inland Waters..

[20] Chang JX (2015). Impact of climate change and human activities on runoff in the weihe river basin, china. Quaternary International..

[21] Guo SL, Guo JL, Hou YK, Hong XJ (2015). Prediction of future runoff change based on budyko hypothesis in yangtze river basin. *Advances In Water*. Science..

[22] Du HM (2014). Runoff variation in minjiang river basin and its response to climate change. Journal of Yibin University..

[23] Jiang Q (2012). An Analysis of runoff changes of tuojiang river basin in the late twentieth century. Journal of Neijiang Normal University..

[24] Yan. B, Xie Y, Zhao C (2018). Analysis of the impact of shifosi reservoir water level on underground water. Journal of Water and Climate Change..

[25] Xu JJ (2008). Spatial and temporal variation of runoff in the yangtze river basin during the past 40 years. Quaternary International.

[26] Qi DM, Zhou CY, Li YQ, Chen YR (2012). Cause analysis of climate changes in southwest china. Plateau and Mountain Meteorology Research..

[27] Chen C, Pang YM, Pan XB (2010). Analysis of variation characteristics of air temperature and precipitation in sichuan basin in recent half century. Chinese Journal of Agrometeorology..

[28] Fu JY, Wang WG (2019). On the lower bound of budyko curve: the influence of precipitation seasonality. Journal of Hydrology..

[29] Yang HB, Yang DW, Lei ZD, Sun FB (2008). New analytical derivation of the mean annual water-energy balance equation. Water Resources Research..

[30] Du QQ (2018). Changes in air temperature of china in response to global warming hiatus. Acta Geographica Sinica..

[31] Zhao FB (2020). Predicting the climate change impacts on water-carbon coupling cycles for a loess hilly-gully watershed. Journal of Hydrology.

[32] Sun PC (2020). Quantifying the contributions of climate variation, land use change, and engineering measures for dramatic reduction in streamflow and sediment in a typical loess watershed, china. Ecological Engineering..

[33] Li ZW (2016). Quantifying the impacts of climate and human activities on water and sediment discharge in a karst region of southwest china. Journal of Hydrology..

[34] Zhao YF (2015). Quantifying the anthropogenic and climatic contributions to changes in water discharge and sediment load into the sea: a case study of the yangtze river, china. Science of the Total Environment..

[35] Wu JW, Miao CY, Zhang XM, Yang TT, Duan QY (2017). Detecting the quantitative hydrological response to changes in climate and human activities. Science of the Total Environment..

[36] Liu XW, Peng DZ, Xu ZX (2017). Identification of the impacts of climate changes and human activities on runoff in the jinsha river basin, china. Advances in Meteorology..

[37] Liu MX, Xu XL, Wang DB, Sun AY, Wang KL (2016). Karst catchments exhibited higher degradation stress from climate change than the non-karst catchments in southwest china: an ecohydrological perspective. Journal of Hydrology..

[38] Zhao FB (2018). Parameter uncertainty analysis of the swat model in a mountain-loess transitional watershed on the chinese loess plateau. Water..

[39] Sun PC (2019). Remote sensing and modeling fusion for investigating the ecosystem water-carbon coupling processes. Science of the total environment..

[40] Zhang SN (2019). Climate change-induced drought evolution over the past 50 years in the southern chinese loess plateau. Environmental Modelling and Software..

[41] Wang H (2017). Impact of lucc on streamflow based on the swat model over the wei river basin on the loess plateau in china. Hydrol Earth Syst..

[42] Wu JW, Miao CY, Wang YM, Duan QY, Zhang XM (2017). Contribution analysis of the long-term changes in seasonal runoff on the loess plateau, china, using eight budyko-based methods. Journal of Hydrology..

[43] Zheng HX (2009). Responses of streamflow to climate and land surface change in the headwaters of the yellow river basin. Water Resources Research..

[44] Zeng SD, Zhan CS, Sun FB, Du H, Wang FY (2015). Effects of climate change and human activities on surface runoff in the luan river basin. Advances in Meteorology..

[45] Yang HB, Yang DW, Hu QF (2014). An error analysis of the budyko hypothesis for assessing the contribution of climate change to runoff. Water Resources Research..

[46] Thavhana MP, Savage MJ, Moeletsi ME (2018). SWAT model uncertainty analysis, calibration and validation for runoff simulation in the luvuvhu river catchment, south africa. Physics and Chemistry of the Earth..

[47] Zhang YY (2018). Simulation and assessment of urbanization impacts on runoff metrics: insights from landuse changes. Journal of Hydrology..

[48] Wang JF, Gao YC, Wang S (2018). Assessing the response of runoff to climate change and human activities for a typical basin in the northern taihang mountain, china. Journal of Earth System Science..

[49] Peng X (2017). Long-term trend in ground-based air temperature and its responses to atmospheric circulation and anthropogenic activity in the yangtze river delta, china. Atmospheric Research..

[50] Chen J (2014). Variability and trend in the hydrology of the yangtze river, china: annual precipitation and runoff. Journal of Hydrology..

[51] Yang XL (2012). Trends in temperature and precipitation in the zhangweinan river basin during the last 53 years. Procedia Environmental Sciences..

[52] Li XH, Zhang Q (2015). Variation of floods characteristics and their responses to climate and human activities in poyang lake, china. Chinese Geographical Science..

[53] Mann H (1945). Nonparametric test against trend. Econometrica..

[54] Kendall, M. Rank correlation measures. (*Charles Griffin*, 1975).

[55] Huang SZ, Chang JX, Huang Q, Chen YT, Leng GY (2016). Quantifying the relative contribution of climate and human impacts on runoff change based on the budyko hypothesis and svm model. Water Resources Management..

[56] Caracciolo D, Pumo D, Viola F (2018). Budyko’s based method for annual runoff characterization across different climatic areas: an application to united state. s. Water Resources Management..

[57] Zhu WB, Jia SF, Lall U, Cao Q, Mahmood R (2019). Relative contribution of climate variability and human activities on the water loss of the chari/logone river discharge into lake chad: a conceptual and statistical approach. Journal of Hydrology..

[58] Zheng YT, Huang YF, Zhou S, Wang KY, Wang GQ (2018). Effect partition of climate and catchment changes on runoff variation at the headwater region of the yellow river based on the budyko complementary relationship. Science of the Total Environment..

[59] Yang DW (2009). Impact of vegetation coverage on regional water balance in the nonhumid regions of china. Water Resources Research..

[60] Xing WQ, Wang WG, Zou S, Deng C (2018). Projection of future runoff change using climate elasticity method derived from budyko framework in major basins across china. Global and Planetary Change..

[61] Wang WG (2016). The analytical derivation of multiple elasticities of runoff to climate change and catchment characteristics alteration. Journal of Hydrology..

[62] Han ZY, Long D, Fang Y, Hou AZ, Hong Y (2019). Impacts of climate change and human activities on the flow regime of the dammed lancang river in southwest china. Journal of Hydrology..

